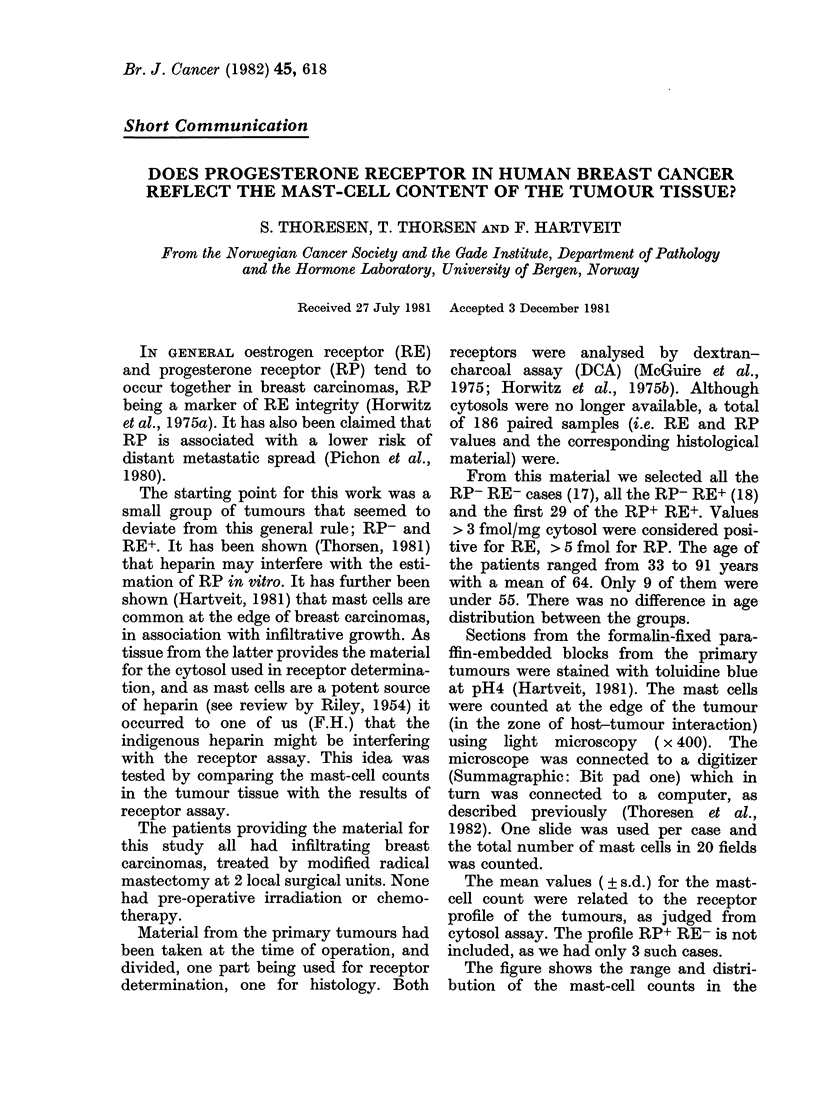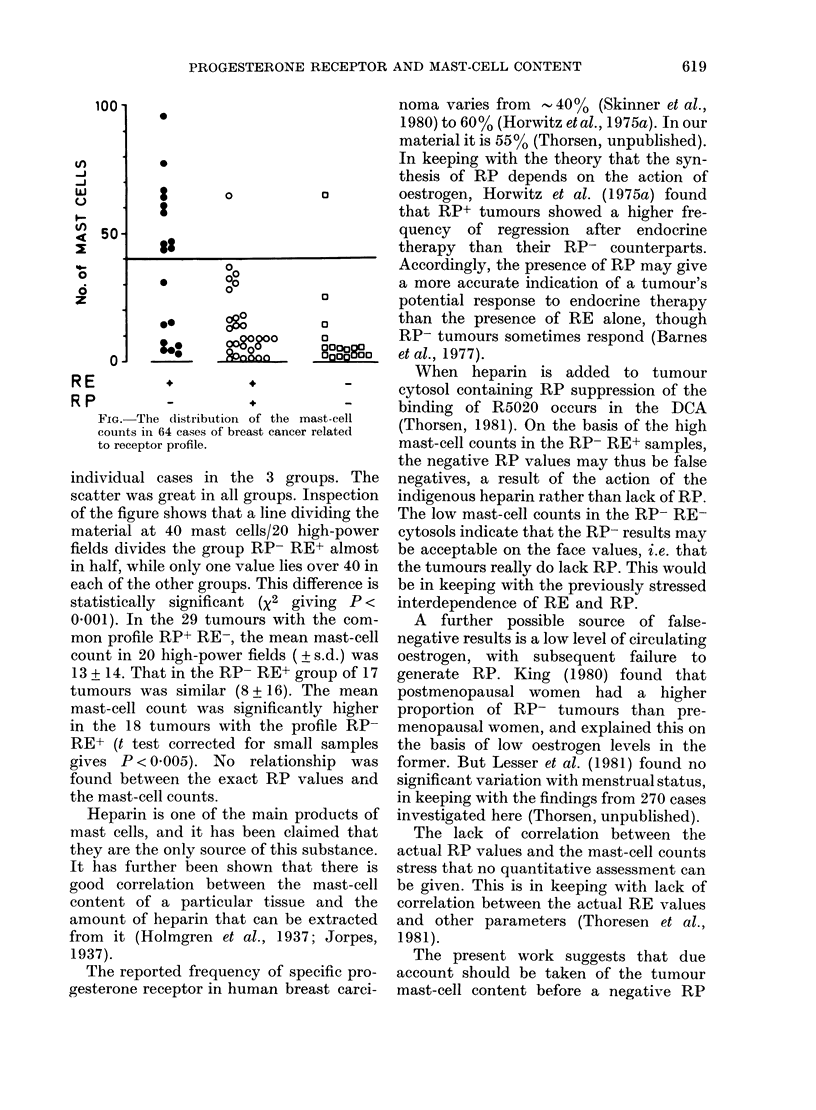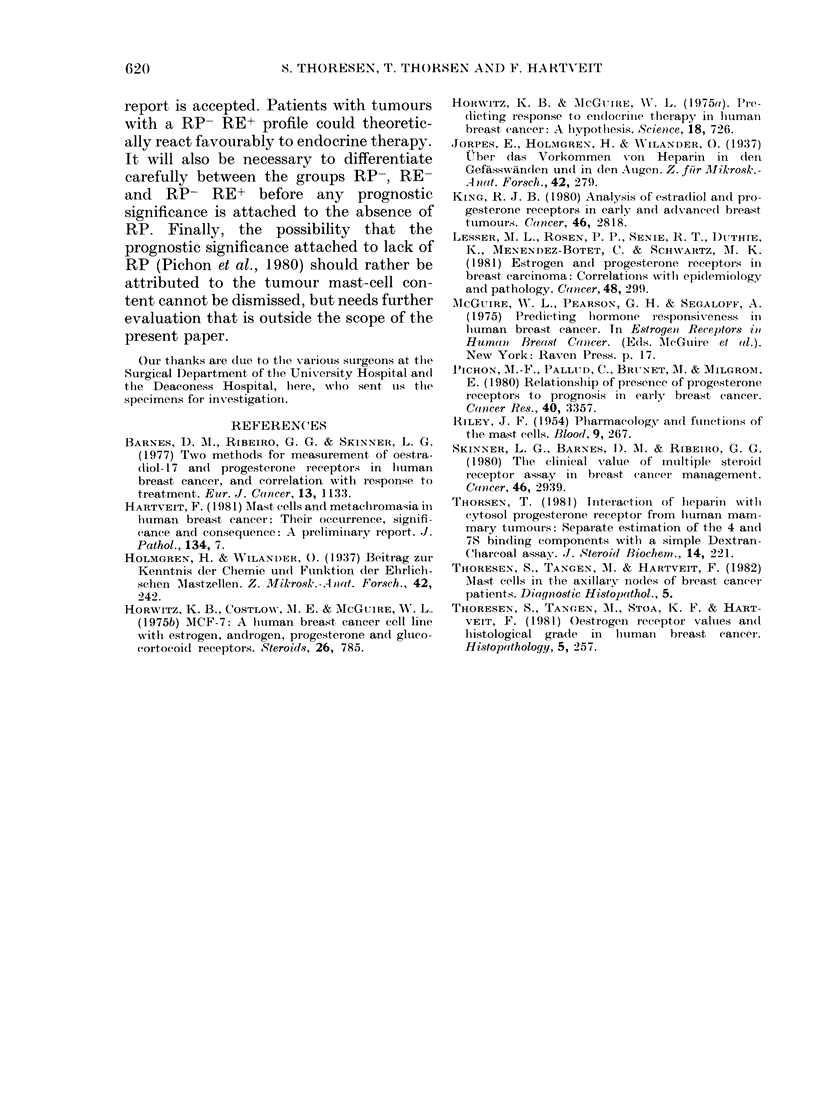# Does progesterone receptor in human breast cancer reflect the mast-cell content of the tumour tissue?

**DOI:** 10.1038/bjc.1982.100

**Published:** 1982-04

**Authors:** S. Thoresen, T. Thorsen, F. Hartveit


Br. J. Cancer (1982) 45, 618

### Short Communication

### DOES PROGESTERONE RECEPTOR IN HUMAN BREAST CANCER REFLECT THE MAST-CELL CONTENT OF THE TUMOUR TISSUE?

S. THORESEN, T. THORSEN AND F. HARTVEIT

*From the Norwegian Cancer Society and the Gade Institute, Department of Pathology and the Hormone Laboratory, University of Bergen, Norway*

Received 27 July 1981 Accepted 3 December 1981

IN GENERAL oestrogen receptor (RE) and progesterone receptor (RP) tend to occur together in breast carcinomas, RP being a marker of RE integrity (Horwitz *et al.*, 1975*a*). It has also been claimed that RP is associated with a lower risk of distant metastatic spread (Pichon *et al.*, 1980).

The starting point for this work was a small group of tumours that seemed to deviate from this general rule; $RP^-$ and $RE^+$. It has been shown (Thorsen, 1981) that heparin may interfere with the estimation of RP *in vitro*. It has further been shown (Hartveit, 1981) that mast cells are common at the edge of breast carcinomas, in association with infiltrative growth. As tissue from the latter provides the material for the cytosol used in receptor determination, and as mast cells are a potent source of heparin (see review by Riley, 1954) it occurred to one of us (F.H.) that the indigenous heparin might be interfering with the receptor assay. This idea was tested by comparing the mast-cell counts in the tumour tissue with the results of receptor assay.

The patients providing the material for this study all had infiltrating breast carcinomas, treated by modified radical mastectomy at 2 local surgical units. None had pre-operative irradiation or chemotherapy.

Material from the primary tumours had been taken at the time of operation, and divided, one part being used for receptor determination, one for histology. Both receptors were analysed by dextran–charcoal assay (DCA) (McGuire *et al.*, 1975; Horwitz *et al.*, 1975*b*). Although cytosols were no longer available, a total of 186 paired samples (*i.e.* RE and RP values and the corresponding histological material) were.

From this material we selected all the $RP^-$ $RE^-$ cases (17), all the $RP^-$ $RE^+$ (18) and the first 29 of the $RP^+$ $RE^+$. Values $> 3$ fmol/mg cytosol were considered positive for RE, $> 5$ fmol for RP. The age of the patients ranged from 33 to 91 years with a mean of 64. Only 9 of them were under 55. There was no difference in age distribution between the groups.

Sections from the formalin-fixed paraffin-embedded blocks from the primary tumours were stained with toluidine blue at pH4 (Hartveit, 1981). The mast cells were counted at the edge of the tumour (in the zone of host–tumour interaction) using light microscopy ($\times 400$). The microscope was connected to a digitizer (Summagraphic: Bit pad one) which in turn was connected to a computer, as described previously (Thoresen *et al.*, 1982). One slide was used per case and the total number of mast cells in 20 fields was counted.

The mean values ($\pm$ s.d.) for the mast-cell count were related to the receptor profile of the tumours, as judged from cytosol assay. The profile $RP^+$ $RE^-$ is not included, as we had only 3 such cases.

The figure shows the range and distribution of the mast-cell counts in the

FIG.—The distribution of the mast-cell counts in 64 cases of breast cancer related to receptor profile.

individual cases in the 3 groups. The scatter was great in all groups. Inspection of the figure shows that a line dividing the material at 40 mast cells/20 high-power fields divides the group $RP^-$ $RE^+$ almost in half, while only one value lies over 40 in each of the other groups. This difference is statistically significant ($\chi^2$ giving $P <$ 0·001). In the 29 tumours with the common profile $RP^+$ $RE^-$, the mean mast-cell count in 20 high-power fields ($\pm$ s.d.) was $13 \pm 14$. That in the $RP^-$ $RE^+$ group of 17 tumours was similar ($8 \pm 16$). The mean mast-cell count was significantly higher in the 18 tumours with the profile $RP^-$ $RE^+$ ($t$ test corrected for small samples gives $P < 0{\cdot}005$). No relationship was found between the exact RP values and the mast-cell counts.

Heparin is one of the main products of mast cells, and it has been claimed that they are the only source of this substance. It has further been shown that there is good correlation between the mast-cell content of a particular tissue and the amount of heparin that can be extracted from it (Holmgren *et al.*, 1937; Jorpes, 1937).

The reported frequency of specific progesterone receptor in human breast carcinoma varies from ~40% (Skinner *et al.*, 1980) to 60% (Horwitz *et al.*, 1975*a*). In our material it is 55% (Thorsen, unpublished). In keeping with the theory that the synthesis of RP depends on the action of oestrogen, Horwitz *et al.* (1975*a*) found that $RP^+$ tumours showed a higher frequency of regression after endocrine therapy than their $RP^-$ counterparts. Accordingly, the presence of RP may give a more accurate indication of a tumour's potential response to endocrine therapy than the presence of RE alone, though $RP^-$ tumours sometimes respond (Barnes *et al.*, 1977).

When heparin is added to tumour cytosol containing RP suppression of the binding of R5020 occurs in the DCA (Thorsen, 1981). On the basis of the high mast-cell counts in the $RP^-$ $RE^+$ samples, the negative RP values may thus be false negatives, a result of the action of the indigenous heparin rather than lack of RP. The low mast-cell counts in the $RP^-$ $RE^-$ cytosols indicate that the $RP^-$ results may be acceptable on the face values, *i.e.* that the tumours really do lack RP. This would be in keeping with the previously stressed interdependence of RE and RP.

A further possible source of false-negative results is a low level of circulating oestrogen, with subsequent failure to generate RP. King (1980) found that postmenopausal women had a higher proportion of $RP^-$ tumours than premenopausal women, and explained this on the basis of low oestrogen levels in the former. But Lesser *et al.* (1981) found no significant variation with menstrual status, in keeping with the findings from 270 cases investigated here (Thorsen, unpublished).

The lack of correlation between the actual RP values and the mast-cell counts stress that no quantitative assessment can be given. This is in keeping with lack of correlation between the actual RE values and other parameters (Thoresen *et al.*, 1981).

The present work suggests that due account should be taken of the tumour mast-cell content before a negative RP

report is accepted. Patients with tumours with a $RP^-$ $RE^+$ profile could theoretically react favourably to endocrine therapy. It will also be necessary to differentiate carefully between the groups $RP^-$, $RE^-$ and $RP^-$ $RE^+$ before any prognostic significance is attached to the absence of RP. Finally, the possibility that the prognostic significance attached to lack of RP (Pichon *et al.*, 1980) should rather be attributed to the tumour mast-cell content cannot be dismissed, but needs further evaluation that is outside the scope of the present paper.

Our thanks are due to the various surgeons at the Surgical Department of the University Hospital and the Deaconess Hospital, here, who sent us the specimens for investigation.

### REFERENCES

BARNES, D. M., RIBEIRO, G. G. & SKINNER, L. G. (1977) Two methods for measurement of oestradiol-17 and progesterone receptors in human breast cancer, and correlation with response to treatment. *Eur. J. Cancer*, **13**, 1133.

HARTVEIT, F. (1981) Mast cells and metachromasia in human breast cancer: Their occurrence, significance and consequence: A preliminary report. *J. Pathol.*, **134**, 7.

HOLMGREN, H. & WILANDER, O. (1937) Beitrag zur Kenntnis der Chemie und Funktion der Ehrlichschen Mastzellen. *Z. Mikrosk.-Anat. Forsch.*, **42**, 242.

HORWITZ, K. B., COSTLOW, M. E. & MCGUIRE, W. L. (1975*b*) MCF-7: A human breast cancer cell line with estrogen, androgen, progesterone and glucocortocoid receptors. *Steroids*, **26**, 785.

HORWITZ, K. B. & MCGUIRE, W. L. (1975*a*). Predicting response to endocrine therapy in human breast cancer: A hypothesis. *Science*, **18**, 726.

JORPES, E., HOLMGREN, H. & WILANDER, O. (1937) Über das Vorkommen von Heparin in den Gefässwänden und in den Augen. *Z. für Mikrosk.-Anat. Forsch.*, **42**, 279.

KING, R. J. B. (1980) Analysis of estradiol and progesterone receptors in early and advanced breast tumours. *Cancer*, **46**, 2818.

LESSER, M. L., ROSEN, P. P., SENIE, R. T., DUTHIE, K., MENENDEZ-BOTET, C. & SCHWARTZ, M. K. (1981) Estrogen and progesterone receptors in breast carcinoma: Correlations with epidemiology and pathology. *Cancer*, **48**, 299.

MCGUIRE, W. L., PEARSON, G. H. & SEGALOFF, A. (1975) Predicting hormone responsiveness in human breast cancer. In *Estrogen Receptors in Human Breast Cancer*. (Eds. McGuire *et al.*). New York: Raven Press. p. 17.

PICHON, M.-F., PALLUD, C., BRUNET, M. & MILGROM, E. (1980) Relationship of presence of progesterone receptors to prognosis in early breast cancer. *Cancer Res.*, **40**, 3357.

RILEY, J. F. (1954) Pharmacology and functions of the mast cells. *Blood*, **9**, 267.

SKINNER, L. G., BARNES, D. M. & RIBEIRO, G. G. (1980) The clinical value of multiple steroid receptor assay in breast cancer management. *Cancer*, **46**, 2939.

THORSEN, T. (1981) Interaction of heparin with cytosol progesterone receptor from human mammary tumours: Separate estimation of the 4 and 7S binding components with a simple Dextran-Charcoal assay. *J. Steroid Biochem.*, **14**, 221.

THORESEN, S., TANGEN, M. & HARTVEIT, F. (1982) Mast cells in the axillary nodes of breast cancer patients. *Diagnostic Histopathol.*, **5**.

THORESEN, S., TANGEN, M., STØA, K. F. & HARTVEIT, F. (1981) Oestrogen receptor values and histological grade in human breast cancer. *Histopathology*, **5**, 257.